# Effects of Low ω6:ω3 Ratio in Sow Diet and Seaweed Supplement in Piglet Diet on Performance, Colostrum and Milk Fatty Acid Profiles, and Oxidative Status

**DOI:** 10.3390/ani10112049

**Published:** 2020-11-05

**Authors:** Thi Xuan Nguyen, Alessandro Agazzi, Marcello Comi, Valentino Bontempo, Invernizzi Guido, Sara Panseri, Helga Sauerwein, Peter David Eckersall, Richard Burchmore, Giovanni Savoini

**Affiliations:** 1 Department of Health, Animal Science and Food Safety ‘Carlo Cantoni’ (VESPA), Università degli Studi di Milano, Via dell’ Università 6, 26900 Lodi, Italy; alessandro.agazzi@unimi.it (A.A.); valentino.bontempo@unimi.it (V.B.); guido.invernizzi@unimi.it (I.G.); sara.panseri@unimi.it (S.P.); giovanni.savoini@unimi.it (G.S.); 2 College of Medical, Veterinary and Life Sciences, School of Veterinary Medicine, University of Glasgow, Garscube Estate, Switchback Road, Bearsden G61 1QH, UK; David.Eckersall@glasgow.ac.uk (P.D.E.); Richard.Burchmore@glasgow.ac.uk (R.B.); 3 Department of Human Science and Quality of Life Promotion, Università Telematica San Raffaele Roma, Via di Val Cannuta 247, 00166 Rome, Italy; marcello.comi@uniroma5.it; 4 Institute of Animal Science, Physiology and Hygiene Unit, University of Bonn, 53115 Bonn, Germany; sauerwein@uni-bonn.de

**Keywords:** sow, ω6:ω3 polyunsaturated fatty acids, seaweed, piglet

## Abstract

**Simple Summary:**

Feeding maternal animals divergent ratios of omega-6 (ω6) and omega-3 (ω3) fatty acids can change not only their health, physiological condition, and performance but also do the same for their offspring. In swine production, various ω6:ω3 ratios have been tested, but the search for an optimal proportion in the sow diet is still in progress. For piglets, weaning oxidative stress has been alleviated by supplementing with abundant sources of bioactive compounds. In this case, brown seaweed, a rich source of natural antimicrobials and antioxidants, can be a good candidate, but its supplementation in piglet diet is limited. This study explores the hypothesis that feeding a low ω6:ω3 ratio diet to sows during gestation and lactation, together with the supplementation of *Ascophyllum nodosum* for piglets during the post-weaning period, could benefit piglets’ performance and oxidative status more than the respective single treatment provided to the mother or the piglet. Results showed that the low dietary ω6:ω3 ratio (4:1) and seaweed supplement did not affect the post-weaning piglets’ growth rate and oxidative status. However, a low ω6:ω3 ratio diet alone improved weaning survival rate, suckling piglets’ weight gain, and total ω3 fatty acids in colostrum and milk.

**Abstract:**

The ratio of omega-6 (ω6) to omega-3 (ω3) polyunsaturated fatty acids (PUFAs) in the diet contributes to animal health and performance modulations because they have mostly opposite physiological functions. Increasing ω3 PUFAs content in the maternal diet can stimulate antioxidative capacity in sow and piglets; however, the optimal ratio of ω6 and ω3 PUFAs in the sow diet is still under discussion. Rich sources of bioactive constituents such as brown seaweed are an excellent supplementation to promote animal health and antioxidant status. However, the knowledge of the effects of this compound, specifically in post-weaning piglets, is still limited. Moreover, the combined effect of a low ω6:ω3 PUFAs ratio in sow diet and seaweed supplementation in post-weaning piglets’ diet has never been studied. This research aims to assess the combined effect of a low ω6:ω3 ratio in sow diets and seaweed supplementation in piglet diets on their growth and oxidative status. We also assessed the impact of a low ω6:ω3 ratio in the maternal diet on reproduction, milk fatty acid (FA) profile, and plasma leptin concentration. Two sow diets (*n* = 8 each) contained either a control ratio (CR, 13:1 during gestation, starting from day 28 (G28) and 10:1 during lactation) or a low ratio (LR, 4:1 from G28 until the end of lactation (L-End)) of ω6:ω3 FA by adding soybean oil or linseed oil, respectively. Reproductive performance was evaluated. Colostrum and milk at lactation day 7 (L7) and L-End were collected to analyze FA profile. Plasma was collected at G28, G79, G108, L7, L14, and L-End for determination of leptin and oxidative status. At weaning, 20 male piglets were selected per sow group to form 4 diet treatments (*n* = 10 each), which were supplemented with or without 4 g/kg seaweed. Recording of growth performance and collection of blood were performed at days 0, 7, 15, and 21 of post-weaning for oxidative status. LR diet increased (*p* < 0.05) the survival rate of piglets at weaning, and individual and litter weight gains. Colostrum and milk at L7 and L-End had lower (*p* < 0.05) ω6:ω3 ratio in LR sows. Interaction between dietary treatments on sows and piglets was revealed for all examined growth parameters at most time points (*p* < 0.05). LR diet did not affect plasma leptin levels and oxidative status. These findings suggest that the seaweed supplement during post-weaning could not improve growth rate and oxidative status of piglets born from mothers receiving a low dietary ω6:ω3 ratio (4:1) during gestation and lactation. However, this low ratio was beneficial for weaning survival rate, sucking piglets’ weight gain, and ω3 enrichment in colostrum and milk.

## 1. Introduction

In pig production, there is a growing interest in lowering the ratio of omega-6 (ω6):omega-3 (ω3) polyunsaturated fatty acids (PUFAs) below 10:1 in maternal diets by using divergent sources of ω3 PUFAs (e.g., fish oil, linseed oil) to promote the health and performance of sows and piglets [1,2,3,4,5]. The enrichment of ω3 PUFAs in the diet has been shown to stimulate the antioxidative capacity in sow plasma and glutathione peroxidase activity (GSH-Px, an enzyme protecting cells against oxidative damage) in piglet liver, and decreased lipid peroxidation in piglet plasma [6]. A previous study found that increasing ω3 PUFAs in the maternal diet enhanced anti-inflammatory properties and reduced the pro-inflammatory effects of ω6 PUFAs, thus maintaining homeostasis [7]. A recent meta-analysis supports this notion in human studies [8], which showed that increasing the portion of ω3 PUFAs in the diet enhances the antioxidant defense mechanisms. However, there continues to be a debate about the optimal ratio of ω6 and ω3 PUFAs in the sow diet and how these effects can be transmitted from mother to offspring via milk, especially effects on piglets’ performance and oxidative status.

For piglets, weaning is the most stressful event in life: the transition from a milk-based to a solid diet and changes in the environment and social relationships can reduce feed intake and conversion efficiency, disrupting gut mucosal barriers, and leading to reduced growth and health [9]. Moreover, piglets undergo oxidative stress and inflammation after weaning that can further participate in poor performance [10]. These adverse outcomes can be alleviated by supplementing with abundant sources of bioactive compounds [10,11,12,13]. Brown seaweed *Ascophyllum nodosum* (*A. nodosum*) is a rich source of natural antioxidants [14] and antimicrobials [15], which can inhibit or impede oxidative damage by neutralizing free radicals in the cell [16]. Therefore, supplementing *A. nodosum* in the post-weaning diet can promote piglet health and performance. To our knowledge, only a few studies have investigated the effect of intact *A. nodosum* meal on the oxidative status of post-weaning piglets [17,18]. Moreover, none of them examined the possible combined or additive effect of low ω6: ω3 PUFAs ratio in sow diet and *A. nodosum* supplementation in post-weaning piglets’ diet.

Herein, we hypothesized that feeding a low ω6:ω3 ratio diet to sows during gestation and lactation, together with the supplementation of *A. nodosum* for piglets during the post-weaning period could benefit piglets’ performance and oxidative status more than the respective single treatment provided to the mother or the piglet. With this purpose, we also considered the effects of maternal dietary low ω6:ω3 ratio on their reproductive performance, milk fatty acids (FA) profile, regulation of plasma leptin concentrations (which indicates sows’ body fat content), and oxidative status.

## 2. Materials and Methods

### 2.1. Animals and Housing

The sow experiment was conducted on a commercial swine farm (Arioli and Sangalli Agricultural Company S.S., Genzone, Italy). Sows were artificially inseminated with pooled semen (Topdelta boar) and kept in groups from one week after artificial insemination until one week before farrowing. Sixteen multiparous sows had similar body weight (202.57 ± 7.16 kg, mean ± SEM) and body condition score (2.36 ± 0.12, mean ± SEM) at the beginning of the trial. On day 108, gestating sows were moved to individual farrowing crates and stayed there until weaning. Within 24 h of birth, ear notching and tagging, iron injection, needle teeth clipping, and tail docking were performed.

The piglet trial was performed at the Animal Production Research and Teaching Centre, University of Milan (Lodi, Italy). Piglets were weaned at day 26 (±1.76) of age (6.46 ± 0.15 kg of body weight, mean ± SE), and 20 male piglets per dietary treatment were selected from the sows (40 piglets in total). They were housed in individual pens (0.47 m^2^/pen) equipped with a bite nipple drinker and self-feeder. The experimental protocols were approved by the Ethical Committee of the University of Milan (OPBA 67/2018) and the Italian Ministry of Health (authorization n. 168/2019 PR).

### 2.2. Experimental Diets

Sows were randomly allocated to one of two diet treatments that contained either control ratio (CR, 13:1 during gestation, starting from day 28 (G28) and 10:1 during lactation) or a low ratio (LR, 4:1, from G28 until the end of lactation) of ω6:ω3 PUFAs. The ω6 and ω3 fatty acids for this study were derived from soybean oil and linseed oil (Mazzoleni s.pa., Bergamo, Italy). The ω6 and ω3 PUFAs content (per total fatty acids) of soybean oil was 54.3% and 8.5%, (ω6:ω3 = 6.26), of linseed oil was 16.2% and 52.9% (ω6:ω3 = 0.31). Experimental diets were calculated to be isonitrogenous and isoenergetic and to meet the estimated nutrient requirements for sows during gestation and lactation [19], according to the total amount of the basal diet (Table 1) provided to adjust the final ratios of ω6:ω3 PUFAs in the diets to 13:1 during gestation and 10:1 during lactation, and 4:1 from G28 until the end of lactation (Table 2).

Experimental diets (fed as a liquid feed by mixing with water) were supplied from day (d) 28 of gestation until the end of lactation. Soybean oil and linseed oil were added to the barley meal at a rate of 10% to create a mixture before the daily feeding of the sows. The gestation diet was provided at 2.4 kg/d with 15 g/d of soybean or linseed oil from d 28 to d 79 and 2.9 kg/d with 18 g/d of soybean or linseed oil from d 80 to the end of gestation. Sows were fed per pen (8 sows/pen). The lactation diet was fed at 1 kg/d on the farrowing day (d 0) and then gradually increased to a maximum of 7.5 kg/d at weaning. During lactation, soybean oil and linseed oil were added daily to the individually basal diet administered according to the lactation feeding plan. Feed was provided twice a day and sows had unlimited access to freshwater. Feed was offered (increased daily) based on the sows’ feed consumed during the previous day.

For piglets, the meal-based commercial diet (Table 3) was supplemented with or without 4 g/kg seaweed powder *(A. nodosum;* Prodotti Arca S.r.l, Monza, Italy), providing 6.5% crude protein, 3.0% crude fat, 22.5% ash, and 52.5% polysaccharides on an as fed basis. Four groups (*n* = 10 each) were formed: CRCT (maternal ω6:ω3 = 13:1 during gestation and 10:1 during lactation, without seaweed (SW) supplementation); CRSW (ω6:ω3 = 13:1 during gestation and 10:1 during lactation, with SW); LRCT (ω6:ω3 = 4:1 during gestation and lactation, without SW); and LRSW (ω6:ω3 = 4:1 during gestation and lactation, with SW).

### 2.3. Recording and Sampling

The sows’ body weights and body condition scores (BCS) were assessed at the beginning of the trial (G28), middle of gestation (G79), at transferring time to the farrowing crates (G108), and at the end of lactation (L-End). The BCS was estimated using the five points scale (1 = emaciated, 2 = thin, 3 = ideal, 4 = fat, and 5 = obese; [20]). Two sows from the CR group had to be removed from the study because they had early parturition.

Piglets born, born alive, and born dead (stillborn, mummified, crushed, and abnormal) were counted within 24 h postpartum to calculate the survival rate at birth. The number of live piglets was recorded at d 7, d 14, and at weaning to calculate the weaning survival rate. Piglets were weighed at 24 h postpartum, d 7, d 14, and at weaning to calculate average daily weight gain.

Colostrum and milk samples (8–40 mL per sample) were collected after an overnight fast (12 h), by manual milking of all functional mammary glands of each sow within 24 h postpartum and on d 7, d 14, and at weaning. Samples were aliquoted and stored at −80 °C until analysis.

Blood samples were collected at G28, G79, G108, L7, L14, and L-End using ethylenediaminetetraacetic acid (EDTA) tubes and centrifuged (15 min; 3000× *g*; room temperature). The EDTA plasma was aspirated and transferred to storage tubes before being stored at −80 °C until analysis.

For post-weaning piglets, feed supply and residuals per head were recorded every morning, from d 0 (weaning day) until d 21 to calculate average daily feed intake and feed conversion ratio. On d 0, 7, 15, and 21, piglets were weighed, and blood samples were collected from the jugular vein. The EDTA plasma obtained was stored at −80 °C until analysis.

### 2.4. Colostrum and Milk Fatty Acid Analysis

Fat in colostrum and milk was extracted [21] and then derivatized [22]. The resulting fatty acid methyl esters (FAMEs) were measured in a gas chromatographer equipped with a flame ionization detector (Trace^TM^ GC Ultra, Thermo Fisher Scientific S.p.A., Rodano, Milan, Italy). The internal standard was nonadecanoic acid (C19:0; 10 mg/mL of hexane), and the carrier gas was helium (He). FAMEs were separated by a fused silica capillary column (Rt-2560, 100 m × 0.25 mm × 0.25 µm) and followed the given program: 50 °C for 6 min; increased by 10 °C min^−1^ until 170 °C, constant for 30 min; increased by 4 °C min^−1^ to 220 °C, constant for 20 min. Individual FAME was verified by comparing peak retention times with standard mixtures (Supelco 37 FAME Mix, Bellefonte, PA, USA) and pure standard methyl esters from Sigma-Aldrich (Saint Louis, MO, USA).

### 2.5. Leptin and Indicators of Oxidative Status in Plasma

Leptin was measured by an in-house developed ELISA validated for use on porcine samples [23]; the intra- and inter-assay CVs were 7.27 and 8.70%, respectively.

Oxidative damage was measured on levels of hydroperoxides (by-products of free radicals) using the d-ROMs (derivatives of reactive oxygen metabolites) test [24] with modifications [25]. Oxidative damage of proteins was assessed by the AOPP (advanced oxidation products of proteins) assay [26]. The AOPP concentrations are expressed both as molar concentrations and per mg of total protein. Total protein concentrations were measured with the Bradford assay [27]. Oxidative damage of lipids was estimated using the TBARS (thiobarbituric acid reactive substances) assay [28]. The total antioxidative capacity was assessed via the FRAP (ferric reducing ability of plasma) assay [29]. The intra- and inter-assay variations of all four assays were below 8%.

### 2.6. Statistical Analysis

Data relative to the length of gestation and lactation, body weight (BW) and BW gain/loss, reproductive performance, fatty acids (FAs) profile, leptin, and oxidative stress indicators of sows were analyzed using the General Linear Model (GLM) procedure in SAS Studio 3.8, on SAS version 9.04.01M6P11072018 (SAS Institute Inc., Cary, NC, USA). The statistical model considered the ω6:ω3 ratio, day, and their interactions as fixed effects, and individual sows as the repeated effect.

Data on post-weaning piglet performance and oxidative stress indicators were analyzed with a repeated-measures model using a MIXED procedure. The statistical model considered ω6:ω3 ratio, seaweed supplement, day, and their interactions as fixed effects, individual piglets as a repeated effect, and weaning time as a random effect. GENMOD procedure (using generalized estimating equations (GEE) method) was performed to fit the generalized linear models on data that were not normally distributed. This included gain-to-feed ratio of piglets; some FAs (∑SFA, TLA, ∑ω3 and ALA in colostrum; DGLA, EPA, and DHA in milk at d 7 of lactation; GLA, DGLA, ETA, EPA, DHA, and ω6:ω3 ratio in milk at the end of lactation); some oxidative stress indicators in sows (leptin at L7, advanced oxidation products of proteins (AOPP) at G79 and G108, thiobarbituric acid reactive substances (TBARS) at L-End, Ferric reducing ability of plasma (FRAP) at G108 and L7, derivatives of reactive oxygen metabolites (d-ROM) at G28, L7, and L-End) and piglets (AOPP and FRAP at d 7, 15 and 21; TBARS at weaning and d 15; d-ROM from weaning to d 21).

Data are presented as LSM (least square means) ± SEM in tables and LSM ± SE in figures. Figures were plotted using GraphPad Prism version 8.4.2 (GraphPad software, La Jolla, CA, USA). Significance was set at *p* ≤ 0.05.

## 3. Results

### 3.1. Sow Reproductive Performance

Low dietary ω6:ω3 ratio (LR) had no effect on sow body weight, weight gain and loss during gestation and lactation (data unshown). The LR diet did not affect total number of piglets born (LSM ± SE: LR 13.75 ± 0.83 piglets; CR 16.33 ± 0.96 piglets; *p* > 0.05) but decreased the number of born alive by 3.45 piglets compared to CR diet (LR 11.38 ± 0.46 piglets; CR 14.83 ± 0.54 piglets; *p* < 0.001). Total piglets weaned per sow of LR group (10.38 ± 0.57 piglets) and of CR group (11.17 ± 0.65 piglets) were similar (*p* > 0.05). Consequently, the LR diet increased the survival rate of piglets at weaning (LR 90.95 ± 3.66 %; CR 75.95 ± 4.23 %; *p* < 0.05).

**Figure 1:**
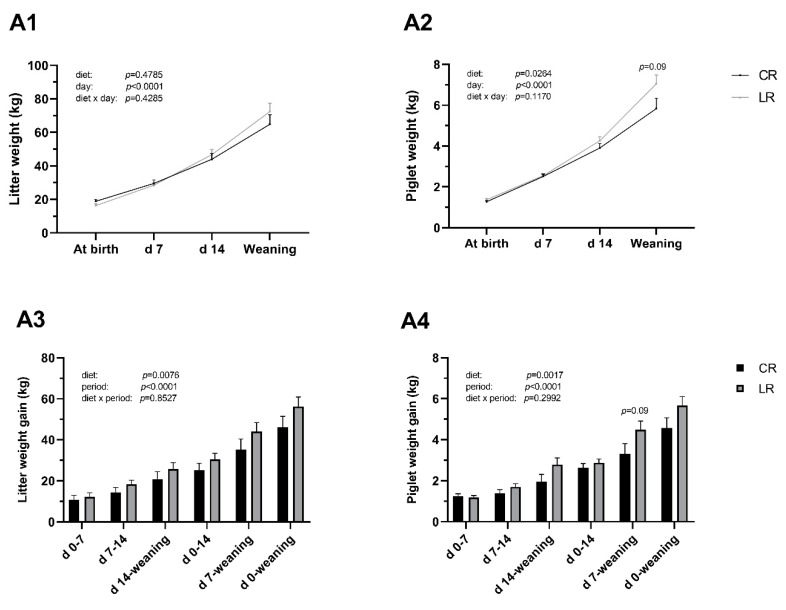
Low dietary ω6:ω3 ratio in sow improves body weight gain of neonatal piglets. Sows were fed diets with ω6:ω3 ratio = 13:1 during gestation, starting from day 28 (G28) and 10:1 during lactation (CR) or ω6:ω3 ratio = 4:1 from G28 until the end of lactation (LR). **A1**: Litter weight development during neonatal period. **A2**: Piglet weight development. **A3**: Litter weight gain per subperiod. **A4**: Piglet weight gain per subperiod. Data are LSM ± SE, *n* = 6 for CR group and *n* = 8 for LR group.

The LR diet increased individual and litter weight gains compared with the CR diet (*p* < 0.05, Figure 1). The LR diet did not affect piglet weight at weaning (LR 7.05 ± 0.44 kg; CR 5.84 ± 0.50 kg; *p* > 0.05) and piglet weight gain from d 7 to weaning (LR 4.49 ± 0.42 kg; CR 3.32 ± 0.49 kg; *p* > 0.05).

### 3.2. Growth Performance of Post-Weaning Piglets

Low maternal dietary ω6:ω3 ratio and seaweed supplementation in the post-weaning piglets’ diet had no influence (*p* > 0.05) on piglet growth (Table 4, Table S1). However, interactions between dietary treatments on sow and piglet were revealed (*p* < 0.05) for BW, average daily gain (ADG), average daily feed intake (ADFI), and gain-to-feed ratio (G:F), mostly related to CRSW vs. CRCT groups. At d 21, BW was higher (*p* < 0.05) in the CRSW than the CRCT group. ADG was improved (*p* < 0.05) during post-weaning. Higher ADFI (*p* < 0.05) was observed during post-weaning for CRSW and LRCT groups compared to LRSW group. G:F ratio was ameliorated (*p* < 0.05) during the first two weeks of post-weaning in CRSW vs. CRCT piglets.

### 3.3. Fatty Acids Composition of Colostrum and Milk

**Figure 2:**
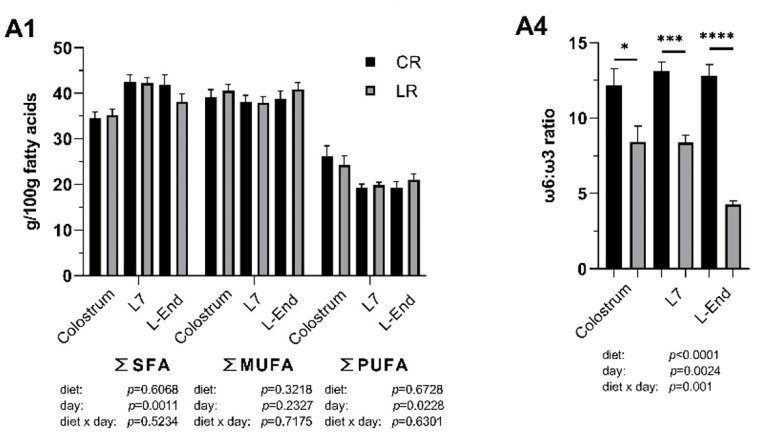

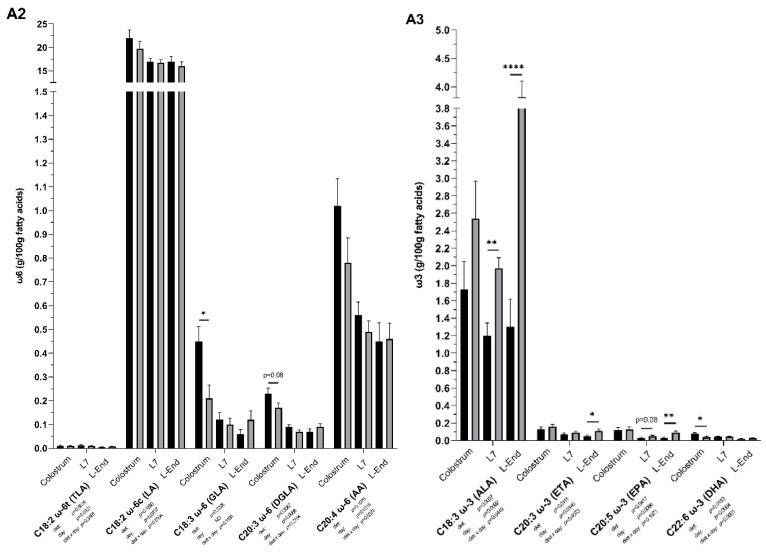
Fatty acid (FA) profile (g/100 g FAs) of colostrum and milk collected on day 7 (L7) and the end of lactation (L-End) from sows fed diets included ω6:ω3 ratio = 13:1 during gestation, starting from day 28 (G28) and 10:1 during lactation (CR) or ω6:ω3 ratio = 4:1 from G28 until L-End (LR). **A1**: SFA, MUFA and PUFA concentrations. **A2**: Individual concentrations of ω6 PUFAs. **A3**: Individual concentrations of ω3 PUFAs. **A4**: ω6:ω3 ratio. SFA = Saturated FAs, MUFA = Monounsaturated FAs, PUFA = Polyunsaturated FAs, TLA = Linoleaidic acid, LA = Linoleic acid, GLA = γ-linolenic acid, DGLA = dihomo-γ-linolenic acid, AA = Arachidonic acid, ALA = α-linolenic acid, ETA = Eicosatrienoic acid, EPA = Eicosapentaenoic acid, DHA = Docosahexaenoic acid. Data are LSM ± SE; *n* = 5, 5 and 6 for CR group and 6, 7, 8 for LR group for colostrum, milk collected at L7 and L-End. * *p* < 0.05, ** *p* < 0.01, *** *p* < 0.001, **** *p* < 0.0001.

Low ω6:ω3 ratio in the maternal diet significantly influenced the fatty acids (FA) profile of both colostrum and milk (Figure 2). In the colostrum, low ω6:ω3 ratio reduced concentrations of γ-linolenic acid (GLA, C18:3 ω6) by two-fold (*p* < 0.05). Low ω6:ω3 ratio also decreased the concentration of docosahexaenoic acid (DHA, C22:6 ω3) by two-fold and the overall ω6:ω3 ratio by one-third (*p* < 0.05). In the milk collected on day 7, low ω6:ω3 ratio increased concentrations of total ω3 FAs and α-linolenic acid (ALA, C18:3 ω3) by 1.6-fold (*p* < 0.01) and consequently decreased the overall ω6:ω3 ratio by one-third (*p* = 0.0001). In the milk collected at the end of lactation, low ω6:ω3 ratio increased concentrations of total ω3 FAs and α-linolenic acid (ALA, C18:3 ω3) by three-fold (*p* < 0.0001), increased concentration of eicosatrienoic acid (ETA, C20:3 ω3) by two-fold (*p* < 0.05), and also increased level of eicosapentaenoic acid (EPA; C20:5 ω3) (*p* < 0.01). Overall, ω6:ω3 ratio was two-thirds declined in the low ratio treatment (*p* < 0.0001). Interactions between sow diet and sampling point were significant in total ω3, α-linolenic acid (ALA, C18:3 ω3) and the overall ω6:ω3 ratio.

### 3.4. Leptin Concentrations and Oxidative Status in Sow Plasma

**Figure 3:**
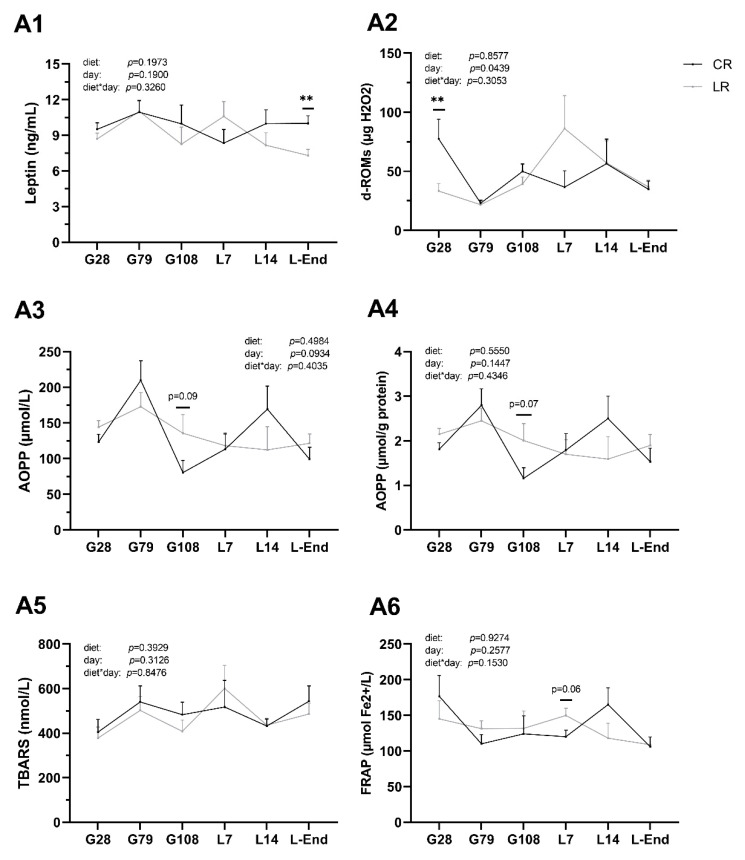
Concentrations of leptin and oxidative stress indicators of sow plasma during gestation and lactation periods. **A1**: Leptin; **A2**: The derivatives of reactive oxygen metabolites (d-ROMs); **A3**, **A4**: Advanced oxidation products of proteins (AOPP); **A5**: Thiobarbituric acid reactive substances (TBARS); **A6**: Ferric reducing ability of plasma (FRAP). CR: sow diet with ω6:ω3 ratio = 13:1 during gestation, starting from day 28 (G28) and 10:1 during lactation; LR: sow diet with ω6:ω3 ratio = 4:1 from G28 until L-End. G: days of gestation, L: days of lactation. Data are LSM ± SE; *n* = 6–8 sows per group. ** *p* < 0.01.

Low dietary ω6:ω3 ratio (LR) had some effects on the concentrations of leptin and oxidative stress indicators in sow plasma (Figure 3). LR diet decreased concentrations of plasma leptin at L-End and d-ROMs at G28 (*p* < 0.01) but had no effect on TBARS, AOPP, and FRAP levels at any time point. Regarding the impact of time on oxidative levels, concentrations of d-ROMs decreased in the middle of gestation and at the end of lactation but increased at the end of gestation and middle of lactation (*p* < 0.05). No interaction between diet and time point was explored for all measured indicators in sow plasma.

### 3.5. Oxidative Status in Plasma of Post-Weaning Piglets

**Figure 4:**
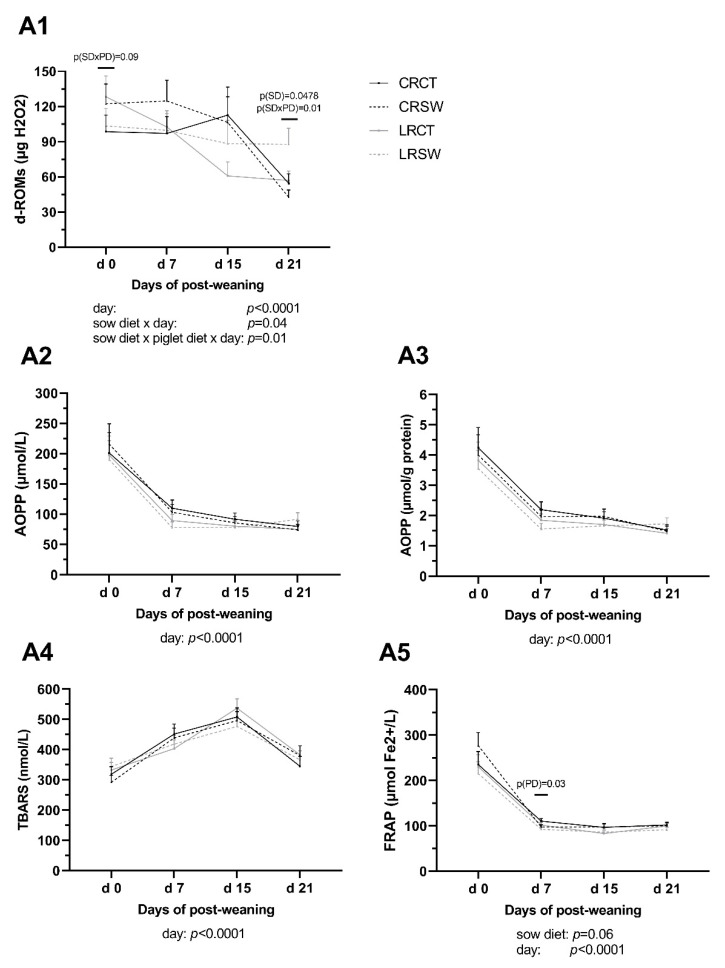
Concentrations of oxidative stress indicators of piglet plasma during the post-weaning period. **A1**: The derivatives of reactive oxygen metabolites (d-ROMs); **A2**: Advanced oxidation products of proteins (AOPP, µmol/L); **A3**: AOPP (µmol/g protein); **A4**: Thiobarbituric acid reactive substances (TBARS); **A5**: Ferric reducing ability of plasma (FRAP). CRCT: piglets fed no seaweed (SW, *Ascophyllum nodosum*), which were nursed by sows fed dietary ω6:ω3 ratio = 13:1 during gestation, starting from day 28 (G28) and 10:1 during lactation. CRSW: piglets fed SW, which were nursed by sows fed dietary ω6:ω3 ratio = 13:1 during gestation and 10:1 during lactation. LRCT: piglets fed no SW, which were nursed by sows fed dietary ω6:ω3 ratio = 4:1 from G28 until the end of lactation. LRSW: piglets fed SW, which were nursed by sows fed dietary ω6:ω3 ratio = 4:1 from G28 until the end of lactation. Data are LSM ± SE; *n* = 10 piglets/group. SD, PD, and SD × PD: effect of sow diet, piglet diet and their interaction between 4 groups at the same sample point.

Low maternal dietary ω6:ω3 ratio (LR) and seaweed supplementation (SW) in the post-weaning piglets’ diet did not affect levels of d-ROMs in CRSW and LRCT piglets at weaning (*p* > 0.05, Figure 4). LR diet increased concentrations of d-ROMs at d 21 (*p* < 0.05): d-ROMs in LRSW piglets was 1.5-fold greater than LRCT and CRCT piglets and was 2-fold higher than CRSW piglets (*p* = 0.01).

SW diet lowered concentration of FRAP at d 7 (*p* < 0.05) but LR diet did not affect levels of FRAP during post-weaning (*p* > 0.05). There were no effects of either sow diet, piglet diet, or their interaction on plasma concentrations of AOPP and TBARS at any time point.

The effects of time on all examined oxidative parameters were significant (*p* < 0.01). Interaction between sow diet and time point, between sow diet, piglet diet and time point were revealed for d-ROMs levels through the post-weaning period (*p* < 0.05).

## 4. Discussion

In this study, we examined whether a low ω6:ω3 ratio (LR) in the sow diet and the supplementation of *A. nodosum* for piglets during the post-weaning period can benefit piglet performance and oxidative status more than the respective single treatment provided to the mother or the piglet. We also considered the effects of low ω6:ω3 ratio in the sows’ diet on reproduction, milk FA profile, plasma leptin concentrations, and oxidative status. We found that the LR diet enhanced total ω3 FA in colostrum and milk, weaning survival rate, and suckling piglets’ weight gain. The interaction between CR and SW diets enhanced piglets’ growth. But LR and SW diets did not affect growth and oxidative status in sows and post-weaning piglets.

We observed that LR diet increased weaning survival rate and sucking piglets’ weight gain compared with the control diet. The enhancement of total ω3 PUFAs in the sows’ colostrum and milk in LR diet could lead to a positive modulation of the intestinal microflora, thus promoting the intestinal health in suckling piglets by inhibiting Toll-like receptor 4 (TLR4) and signaling to reduce inflammation [30]. Our results support previous research findings that link low ω6:ω3 ratio in the maternal diet with piglet growth improvement. Specifically, a trend in improving piglets’ growth rate in the first week of life was reported when a ω6:ω3 ratio of 5:1 was provided to sows compared to an 11:1 ratio [2]. Similarly, lowering dietary ω6:ω3 ratio from 20:1 and 15:1 to 10:1 increased piglet body weight and litter ADG at weaning [3]. Moreover, a 3:1 diet tended to increase litter size on the second and third weeks of life while a 9:1 diet tended to increase litter ADG in the first 14 days of age compared with a 13:1 diet [1]. In our study, LR sows had fewer piglets born alive than CR sows, but their performance was similar to previous research when sows were fed low ω6:ω3 ratios (from 1:1 to 5:1) [31]. The reduction in the number of piglets born alive on the LR diet is difficult to explain: it is not solely due to low dietary ω6:ω3 ratio, but also some unaccounted factors might be involved in the obtained results. The total number of piglets born and the number of born alive are affected by genetic factors of both sows and piglets [32]. However, these traits’ heritability is low (ranging from 0.16 to 0.19 and 0.04 to 0.29, respectively) [33,34]. Therefore, they are strongly affected by the environment. The low heritability of these parameters could explain for their variability between the sows and between CR and LR groups, although we kept these two groups in the same environmental conditions during the feeding trial.

To our knowledge, this is the first study that accounts for a possible combined positive effect of ω3 enriched maternal diet and seaweed supplementation in the post-weaning piglets’ diet on their growth and oxidative status. Results showed that seaweed (*A. nodosum)* supplementation in post-weaning piglets increased BW, ADG, ADFI, and G:F when born from control sow compared with non-seaweed supplemented piglets. *A. nodosum* is considered to be a source of ω3 PUFAs with a high concentration of EPA (C20:5 ω3) and low ω6:ω3 ratio of 2.75 [35]. Thus, this can add more ω3 PUFAs and lower ω6:ω3 ratios in piglets, which were born from control sows and received the seaweed diet. However, this positive result of additional ω3 PUFAs from *A. nodosum* was not seen in piglets born from sows fed ω3 enriched diet. Thus, the combined effect of LR and SW diets is difficult to explain. The literature reports that offering too low a ratio of ω6:ω3 (0.38) to pigs (from growing to finishing) did not affect their growth performance, although it improved total ω3 PUFAs (including ALA, EPA, and DHA) and reduced ω6:ω3 ratio in pork [36].

Lowering the ratio of ω6:ω3 PUFAs by adding linseed oil to the maternal diet can decrease ω6:ω3 ratio in colostrum and milk by enriching ALA concentration [1], thus, increasing concentrations of ALA in piglet liver, brain, and muscle tissues [37], plasma, carcass [38], and intestinal mucosa [39]. Our results are consistent with the findings of Yao et al. [1] when offering high ALA combined with the low LA diet.

However, high ALA intake was found not to affect the DHA level in piglet liver [6] or even decreased DHA concentration in human red blood cells, although it increased EPA level [40]. In line with recent research, the low concentration of DHA in our study was due to the inefficient conversion of ALA to EPA (i.e., 0.2 ~ 5%) and DHA (i.e., 0.05–0.5%) [41,42], which may be explained by several reasons: First, the high rate (59%) of ALA was β-oxidized for the whole body utilization [43]. Second, the lack of elongase-2 expression to elongate C22:5 ω3 to C24:5 ω3 [44], which supports the EPA generation. Third, the competition between LA and ALA for enzyme Δ-6 desaturase, which has a higher affinity for ALA; thus, high LA intake prevents the conversion from ALA to EPA [41,45]. Collectively, our results demonstrate that a linseed oil enriched ω3 diet is a substantial source for endogenous ALA rather than for endogenous DHA concentrations.

Leptin is an important adipocyte-secreted hormone regulating appetite, body weight, reproduction, energy homeostasis, and acts as a pro-inflammatory cytokine [46]. The ω3 enriched diet can regulate leptin in vivo [47]. It tended to decrease leptin in adipose tissue [48], and lower plasma leptin level [49]. In this study, the reducing effect of ω3 enriched diet on plasma leptin in sows at the end of lactation might go with increased β-oxidation of FAs in the liver and skeletal muscle (to a smaller extent) [50].

Pregnancy, delivery process, and lactation are all associated with elevated levels of oxidative stress due to intensive metabolism and lipid peroxidation [51]. We found that a low ratio of ω6:ω3 PUFAs in the maternal diet tended to increase AOPP concentrations at the end of gestation, decreased the number of piglets born and piglets born alive, which could be explained by the involvement of AOPP levels in embryonic mortality [52]. However, the increased level of AOPPs in LR sows did not seem to cause intracellular ROS (H_2_O_2_) production because d-ROMs levels were the same between LR and CR sows at the end of gestation. In this study, ω3 enriched diet did not influence lipid peroxidation of sow plasma. It might be that the malondialdehyde measured in the TBARS test was absorbed quickly in the liver before being eliminated in urine without influencing TBARS levels in blood circulation [53]. Here, we observed that FRAP values of LR sows were similar at the end of gestation and tended to increase at the beginning of lactation, compared with CR sows. The hypothesis may explain that when protein oxidation increased, the sow’s antioxidant system was stimulated and led to FRAP concentrations increasing when sows were offered more ω3 PUFAs.

Weaning is a stressful event for piglets, challenging for health and growth due to various stressors [54]. The post-weaning period is associated with increased oxidative products in plasma: the highest ROM found in the first-week post-weaning was linked to reduced growth rates [55]; NO and H_2_O_2_ were highest at weaning and decreasing after weaning [56]. Similar results were observed in our study.

We could not see any effects of the low ratio of ω6:ω3 PUFAs in the maternal diet and seaweed supplementation in the piglets’ diet on the piglets’ oxidative status. However, the higher ADG and G:F ratio of the LRCT and CRSW piglets indicated benefits of ω3 enriched diet and seaweed supplementation on stimulating antioxidants to reduce excessive ROS production, therefore, improving piglets’ growth performance. Growth performance reflects overall influences over a period, whereas plasma measurements are a single test at a time point [57]. Moreover, this improvement can be explained by the bioactivity of ω3 PUFAs in the maternal diet and seaweed-derived elements (i.e., ascophyllan, laminarin, fucoidans, and phlorotannins) in the post-weaning piglet diet [58]. Maternal diets enriched in ω3 PUFAs with optimal ω6:ω3 ratio might regulate progeny immune response and improve anti-inflammatory activity against pathogens [59], especially the inflammatory response causing by post-weaning stress [4]. Post-weaning diets supplemented with *A. nodosum* can promote piglet intestinal health through its immunomodulatory, anti-inflammatory, antioxidant, and antimicrobial properties [60,61,62], thereby improving piglets’ growth rates.

Overall, our study showed that the ω3 enriched diet during gestation and lactation did not affect the oxidative stress of sows and their post-weaning piglets. Similar results were reported when feeding a dietary ω6:ω3 ratio of 4:1 or 6:1 to sows [6] or feeding a dietary ω6:ω3 ratio of 5:1 to piglets (10.6 kg) [63]. These outcomes may be explained by low double bonds of ALA and its poor conversion rate to EPA and DHA, as shown in the FA profile of colostrum and milk in this study. The dietary ω6:ω3 ratio above 3:1 is considered safe to protect the animal from oxidative damage [5]. Our results suggest that not only the dietary ratio between ω6 and ω3 but also the type of fat source (total double bonds) have synergic effects on the oxidative stress level of the sows and piglets.

## 5. Conclusions

The current study showed that low dietary ω6:ω3 ratio in sow diet during gestation and lactation combined with the seaweed supplementation in piglets after weaning could not improve piglets’ growth rate or antioxidant status. However, the sows’ low ω6:ω3 ratio diet alone improved weaning survival rate, suckling piglets’ weight gain, and total ω3 PUFAs (mainly ALA) in the colostrum and milk. Seaweed supplementation improved growth performance of piglets born from sows which received the higher dietary ω6:ω3 ratio. These findings outline that maternal dietary interventions can significantly affect the progeny, and that seaweed supplementation is effective in improving performance in post-weaning piglets. However, interaction among these treatments remains unclear. Further studies are necessary to enhance the knowledge of maternal dietary interventions during gestation and lactation, and their effect on the progeny or to outline the efficacy of natural antimicrobials and antioxidants in the diets of piglets, also considering omics and epigenetic approaches.

## Figures and Tables

**Table 1:** Composition of Basal Sow Diets.

Item	Gestation	Lactation
**Ingredients (g/kg as fed basis)**		
Corn	284.60	249.10
Barley	224.20	216.70
Wheat bran	208.00	115.80
Distillers grains	125.00	40.00
Biscuit	50.20	52.10
Rice	35.00	35.00
Commercial concentrate *	25.00	250.00
Soybean oil	12.90	14.00
Fish meal	-	14.50
Mineral-vitamin premix **	20.00	11.70
HCl-Lysine	11.20	15.00
**Composition (% DM)**		
Crude protein	15.85	19.92
Crude fat	4.55	4.93
Crude fiber	5.69	5.66
Ash	5.68	4.46
Ca	1.70	1.21
P	0.56	0.57
Ca/P	3.04	2.12
Lysine	1.04	1.34
Methionine	0.18	0.22
Met +Cis	0.37	0.50

* Providing (as fed basis): 32.36% crude protein, 6.80% crude fat, 6.77% crude fiber, 0.80% Na, 2.43% lysine, 0.56% methionine. ** Providing (per kg of complete diet): vitamin A, 10,000 IU; vitamin D_3_, 2000 IU; vitamin E, 48 IU; vitamin K_3_, 1.5 mg; riboflavin, 6 mg; niacin, 40 mg; biotin, 0.2 mg; d-pantothenic, 17 mg; folic acid, 2 mg; choline, 166 mg; vitamin B_6_, 2 mg; and vitamin B_12_, 28 mg. Fe (as FeSO_4_), 90 mg; Cu (as CuSO_4_), 15 mg; Zn (as ZnSO_4_), 50 mg; Mn (as MnO_2_), 54 mg; I (as KI), 0.99 mg; and Se (as Na_2_SeO_3_), 0.25 mg.

**Table 2:** Fatty Acid (g/100 g total fatty acids) of sow diets.

Item	Gestation CR	Gestation LR	Lactation CR	Lactation LR
10:0	0.26	0.22	-	-
12:0	0.35	0.30	1.80	1.56
14:0	0.25	0.21	-	-
16:0	20.23	18.08	17.58	16.06
16:1 ω7	0.50	0.42	-	-
18:0	3.06	3.30	4.36	4.40
18:1 ω9 cis	19.88	19.80	24.51	23.84
18:1 ω7	1.05	1.03	-	0.12
18:2 ω6 cis 9,12	49.79	44.73	46.92	42.90
18:3 ω3	3.72	11.12	4.83	11.12
20:0	0.28	0.24	-	-
20:1 ω9	0.41	0.35	-	-
22:0	0.23	0.20	-	-
ω6	49.79	44.73	46.92	42.90
ω3	3.72	11.12	4.83	11.12
ω6:ω3	13.40	4.02	9.71	3.88

Control ratio (CR): sow diet with ω6:ω3 ratio = 13:1 during gestation, starting from day 28 (G28) and 10:1 during lactation; low ratio (LR): sow diet with ω6:ω3 ratio = 4:1 from G28 until the end of lactation.

**Table 3:** Ingredients and Chemical Composition of the Basal Diet (CT) of post-weaning piglets. Seaweed powder (SW) was added to the basal diet at 4 g/kg feed rate.

Item	Post-Weaning Basal Diet
**Ingredients (g/kg as fed basis)**	
Barley	220.0
Wheat	161.7
Soy protein concentrate (Soicomil R)	98.0
Wheat, flaked	80.0
Corn	80.0
Corn, flaked	60.0
Soybean, meal	59.0
Biscuits	50.0
Whey	50.0
Dextrose, mono	40.0
Barley, flaked	40.0
Soybean oil	20.0
Dicalcium phosphate	10.0
Cocoa oil	10.0
L-Lysine	6.0
DL-Methionine	3.6
L-Threonine	3.5
Sodium chloride	2.7
Vitamin + trace elements	2.5
L-Valine (96.5%)	2.2
L-Tryptophan	0.8
**Composition (%, DM)**	
Dry matter (DM)	89.60
Crude protein	20.10
Crude fat	5.68
Fiber	3.29
Neutral detergent fiber (NDF)	12.91
Acid detergent fiber (ADF)	4.67
Acid detergent lignin (ADL)	0.97
Lysine (Lys), total	1.57
Cystine	0.32
Methionine (Met), total	0.66
Threonine, total	1.09
Tryptophan, total	0.32
Valine	1.08
Phenylalanine	0.85
Tyrosine	0.55
Isoleucine	0.74
Leucine	0.32
Net energy (NE, Mcal/kg)	2.90

**Table 4:** Growth performance of post-weaning piglets fed seaweed (SW).

Sow Diets (SD) Piglet Diets (PD) *	CR		LR		SEM ^1^	*p*-Value		
	CT	SW	CT	SW		SD	PD	SD × PD
No. of piglets **	10	10	10	10				
BW ^2^ (kg)								
d 0	6.19	6.19	6.66	6.36	0.55	ns ^4^	ns	ns
d 7	6.86	7.21	7.58	6.89	0.59	ns	ns	ns
d 15	9.56	10.72	10.62	9.32	0.98	ns	ns	0.018
d 21	12.41 ^b^	14.14 ^a^	13.64	12.13	1.17	ns	ns	0.010
ADG ^2^ (g/d)								
d 0 to 7	51.86	101.30	157.50	100.20	62.74	ns	ns	ns
d 0 to 15	202.76	279.59	275.82	211.51	55.73	ns	ns	0.017
d 0 to 21	276.15 ^b^	358.55 ^a^	345.63	289.71	52.20	ns	ns	0.013
ADFI ^2^ (g/d)								
d 0 to 7	154.46	194.17	222.59	196.79	44.58	ns	ns	ns
d 0 to 15	345.07	394.71	373.65	313.38	56.11	ns	ns	ns
d 0 to 21	458.59	527.04 ^a^	474.17 ^a^	387.27 ^b^	55.81	ns	ns	0.009
G:F ^3^								
d 0 to 7	0.59	0.48	0.70	0.57	0.27	ns	ns	ns
d 0 to 15	0.55 ^b^	0.75 ^a^	0.71	0.67	0.16	ns	ns	0.033
d 0 to 21	0.62	0.73	0.70	0.70	0.08	ns	ns	0.050

CR: sow diet with ω6:ω3 ratio = 13:1 during gestation, starting from day 28 (G28) and 10:1 during lactation; LR: sow diet with ω6:ω3 ratio = 4:1 from G28 until the end of lactation. CT: post-weaning piglets’ diet supplemented without intact seaweed powder. SD: sow diets, PD: piglet diets. BW: body weight, ADG: average daily gain, ADFI: average daily feed intake, G:F: gain-to-feed ratio. * The trial was performed from weaning to day 21 of post-weaning with 4 g seaweed supplementation per kg of feed. Feed residual and daily feed intake of each piglet were recorded every morning, from day 0 (weaning) to day 21 post-weaning to calculate average daily feed intake and feed conversion ratio. Individual body weight of piglets was measured on day 0, 7, 15 and 21 of post-weaning. ** Piglets were kept in individual pens (0.47 m2) with ad libitum feed and water. ^1^ SEM: Standard error of the means. ^a, b^ Means are presented as least square means. ^2^ have normal distribution so mixed procedure was performed. ^3^ do not have normal distribution so GENMOD procedure (using generalized estimating equations (GEE) method) was performed to fit the generalized linear models. ^4^ ns: not significant.

## Supplementary Materials

The following are available online at https://www.mdpi.com/2076-2615/10/11/2049/s1, Table S1: Growth performance of post-weaning piglets fed seaweed (SW).
