# Supplementary material for: Effects of Low ω6:ω3 Ratio in Sow Diet and Seaweed Supplement in Piglet Diet on Performance, Colostrum and Milk Fatty Acid Profiles, and Oxidative Status
Source: Animals (Basel). 2020 Nov 5;10(11):2049. doi: 10.3390/ani10112049 (PMC7694489; doi:10.3390/ani10112049)
Supplement: Supplementary file 1 [file animals-10-02049-s001.pdf]

**Table S1.** Growth performance of post-weaning piglets fed seaweed (SW)

| Sow diets (SD) | CR | | LR | | | *p*-value | | |
|---|---|---|---|---|---|---|---|---|
| **Piglet diets (PD)*** | **CT** | **SW** | **CT** | **SW** | **SEM[1]** | **SD** | **PD** | **SD x PD** |
| No. of piglets** | 10 | 10 | 10 | 10 | | | | |
| BW[2] (kg) | | | | | | | | |
| d 0 | 6.19 | 6.19 | 6.66 | 6.36 | 0.55 | 0.509 | 0.595 | 0.590 |
| d 7 | 6.86 | 7.21 | 7.58 | 6.89 | 0.59 | 0.712 | 0.565 | 0.087 |
| d 15 | 9.56 | 10.72 | 10.62 | 9.32 | 0.98 | 0.852 | 0.886 | 0.018 |
| d 21 | 12.41[b] | 14.14[a] | 13.64 | 12.13 | 1.17 | 0.713 | 0.850 | 0.010 |
| ADG[2] (g/d) | | | | | | | | |
| d 0 to 7 | 51.86 | 101.30 | 157.50 | 100.20 | 62.74 | 0.106 | 0.901 | 0.099 |
| d 7 to 15 | 346.85[b] | 447.64[a] | 375.05[ab] | 298.14[bc] | 60.95 | 0.310 | 0.698 | 0.007 |
| d 15 to 21 | 471.65 | 567.97 | 527.00 | 491.98 | 73.17 | 0.780 | 0.409 | 0.083 |
| d 0 to 15 | 202.76 | 279.59 | 275.82 | 211.51 | 55.73 | 0.955 | 0.824 | 0.017 |
| d 0 to 21 | 276.15[b] | 358.55[a] | 345.63 | 289.71 | 52.20 | 0.993 | 0.616 | 0.013 |
| FI[2] (kg/period) | | | | | | | | |
| d 0 to 7 | 1.08 | 1.36 | 1.56 | 1.38 | 0.31 | 0.123 | 0.757 | 0.152 |
| d 7 to 15 | 3.87 | 4.34 | 4.19 | 3.47 | 0.58 | 0.598 | 0.665 | 0.049 |
| d 15 to 21 | 4.31[b] | 5.00[a] | 4.45[ab] | 3.53[c] | 0.48 | 0.127 | 0.644 | 0.002 |
| d 0 to 15 | 5.18 | 5.92 | 5.60 | 4.70 | 0.84 | 0.596 | 0.851 | 0.060 |
| d 0 to 21 | 9.63[ab] | 11.07[a] | 9.96[a] | 8.13[b] | 1.17 | 0.234 | 0.743 | 0.009 |
| ADFI[2] (g/d) | | | | | | | | |
| d 0 to 7 | 154.46 | 194.17 | 222.59 | 196.79 | 44.58 | 0.123 | 0.757 | 0.152 |
| d 7 to 15 | 483.86 | 542.20 | 523.48 | 433.50 | 72.35 | 0.598 | 0.665 | 0.049 |
| d 15 to 21 | 717.62 | 833.07 | 741.10 | 588.15 | 80.39 | 0.127 | 0.644 | 0.002 |
| d 0 to 15 | 345.07 | 394.71 | 373.65 | 313.38 | 56.11 | 0.596 | 0.851 | 0.060 |
| d 0 to 21 | 458.59[ab] | 527.04[a] | 474.17[a] | 387.27[b] | 55.81 | 0.234 | 0.743 | 0.009 |
| G : F[3] | | | | | | | | |
| d 0 to 7 | 0.59 | 0.48 | 0.70 | 0.57 | 0.27 | 0.377 | 0.198 | 0.960 |
| d 7 to 15 | 0.65 | 0.79 | 0.74 | 0.72 | 0.12 | 0.853 | 0.152 | 0.065 |
| d 15 to 21 | 0.68 | 0.71 | 0.68 | 0.76 | 0.09 | 0.509 | 0.085 | 0.426 |
| d 0 to 15 | 0.55[b] | 0.75[a] | 0.71 | 0.67 | 0.16 | 0.561 | 0.125 | 0.033 |
| d 0 to 21 | 0.62 | 0.73 | 0.70 | 0.70 | 0.08 | 0.457 | 0.068 | 0.050 |

CR: sow diet with ω6:ω3 ratio=13:1 during gestation, starting from day 28 (G28) and 10:1 during lactation; LR: sow diet with ω6:ω3 ratio=4:1 from G28 until the end of lactation. CT: post-weaning piglets dietary supplemented without intact seaweed powder. SD: sow diets, PD: piglet diets. BW: body weight, ADG: average daily gain, ADFI: average daily feed intake, G:F: gain : feed ratio.

* The trial was performed from weaning to d 21 of post-weaning with 4 g seaweed supplementation per kg of feed. Feed residual and daily feed intake of each piglet were recorded every morning, from day 0 (weaning) – 21 post-weaning to calculate average daily feed intake and feed conversion ratio. Individual body weight of piglets was measured on day 0, 7, 15 and 21 of post-weaning.

** Piglets were kept in individual pens (0.47 m2) with *ad libitum* feed and water.

[1] SEM: Standard error of the means. Means are presented as least square means.

[2] have normal distribution so Mixed procedure was performed.

[3] do not have normal distribution so GENMOD procedure (GEE model) was performed.
